# A New Genus, *Luciargentis* gen. nov. Revealed by Morphological and Phylogenetic Evidence in the Family Lecithoceridae from Tibet, China

**DOI:** 10.3390/insects16030242

**Published:** 2025-02-25

**Authors:** Shuai Yu, Haotian Li, Shuxia Wang

**Affiliations:** 1 College of Agriculture and Biology, Liaocheng University, Liaocheng 252000, China; yushuai088@163.com (S.Y.); lihaotian@lcu.edu.cn (H.L.); 2 College of Life Sciences, Nankai University, Tianjin 300071, China

**Keywords:** Crocanthinae, Torodorinae, Lecithocerinae, taxonomy, molecular data

## Abstract

In this study, we describe a new genus, *Luciargentis* Yu and Wang, gen. nov. based on the new species, *Luciargentis obesa* Yu and Wang, sp. nov. Additionally, the genus is placed within the subfamily Crocanthinae, satisfying the criteria of Crocanthinae. We conducted a phylogenetic analysis of the family Lecithoceridae using a concatenated dataset that includes one mitochondrial marker (COI) and six nuclear markers (EF-1α, GAPDH, RpS5, CAD, wingless, and MDH), incorporating 17 exemplars from NCBI and 7 newly sequenced exemplars from this study. The yield tree topologies showed that *Luciargentis obesa* forms a strongly supported monophyletic group with *Crocanthes prasinopis*, a species of Crocanthinae. Moreover, *Luciargentis* + *Crocanthes* were found to be sister groups to Torodorinae. Images of the adults, wing venation, and genitalia of the new species are provided.

## Simple Summary

The family Lecithoceridae is one of the most species-rich groups within Gelechioidea (Lepidoptera), yet it remains relatively underexplored. Currently, it comprises more than 1430 species across four subfamilies. Taxonomic studies of this family primarily relied on morphological data, with molecular data being limited, which hindered the development of its taxonomy. In this study, we try to establish a new genus based on the specimens from Tibet, China and examine its placement within the subfamily Crocanthinae, integrating both morphological and molecular evidence. Furthermore, the new genus provides additional insights that contribute to the ongoing discussion regarding the relationship between the subfamilies Torodorinae and Crocanthinae.

## 1. Introduction

Lecithoceridae, recognized as one of the most species-rich groups within the superfamily Gelechioidea (Lepidoptera), represents the sixth-largest family in this taxonomic group, comprising more than 1430 described species [1,2]. This family is widely distributed across the Oriental, Ethiopian, Australian, and Palaearctic regions. Members of Lecithoceridae can be identified by several distinct external features: antennae that are as long as or longer than the forewing, sub-rectangular or lanceolate forewings, trapezoidal hindwings, and male genitalia typically characterized by a median process of the gnathos that is usually downturned, except in the subfamily Crocanthinae. Additionally, the family still remains relatively underexplored. Park et al. estimated that the actual global species count might be 2–3 times higher than the currently recognized number [1]. Despite its diversity, Lecithoceridae received insufficient scientific attention. This neglect is likely due to its minimal economic significance and the shortage of specialists in the field. Nevertheless, the ecological importance of Lecithoceridae cannot be overlooked. Larvae of this family have been reported to feed on non-living materials and organisms [3,4,5,6], suggesting a significant role in environmental ecosystems.

Lecithoceridae is currently divided into four subfamilies. Three were proposed by Gozmány [3]: (1) Ceuthomadarinae, characterized by the absence of a proboscis; (2) Lecithocerinae, characterized by male genitalia with a costal bar and a short, posteriorly lobed uncus; and (3) Torodorinae, characterized by male genitalia lacking a costal bar but possessing a well-developed, often posteriorly thorned uncus. The fourth subfamily, Crocanthinae, was proposed by Park and is defined by male genitalia with a reduced gnathos [7].

Tibet, often called the “Roof of the World”, lies on the Tibetan Plateau at an average elevation exceeding 4500 m. Renowned for its unique geography, ecology, and environment, Tibet has become a global hotspot for biological research. The region boasts an extraordinary diversity of plant and animal species, many of which are endemic, emphasizing its significance in biodiversity and conservation studies [8]. From 2017 to 2023, our team conducted extensive fieldwork in Tibet, collecting numerous micromoth specimens. Among these, we identified many new taxa, including the species described in this paper. Additionally, Médog, situated in southeastern Tibet, serves as the primary discovery site for the new taxon described in this study. Spanning an area of 34,000 square kilometers with an average elevation of 1200 m, Médog boasts an annual average temperature of 18.4 °C and receives more than 2330 mm of rainfall each year. The region is characterized by its tropical monsoon rainforest and subtropical humid monsoon climate (http://www.motuo.gov.cn, accessed on 10 February 2025).

The aim of this paper is to describe a newly discovered genus, and to discuss its subfamily affiliation within Lecithoceridae.

## 2. Materials and Methods

**Figure 1:**
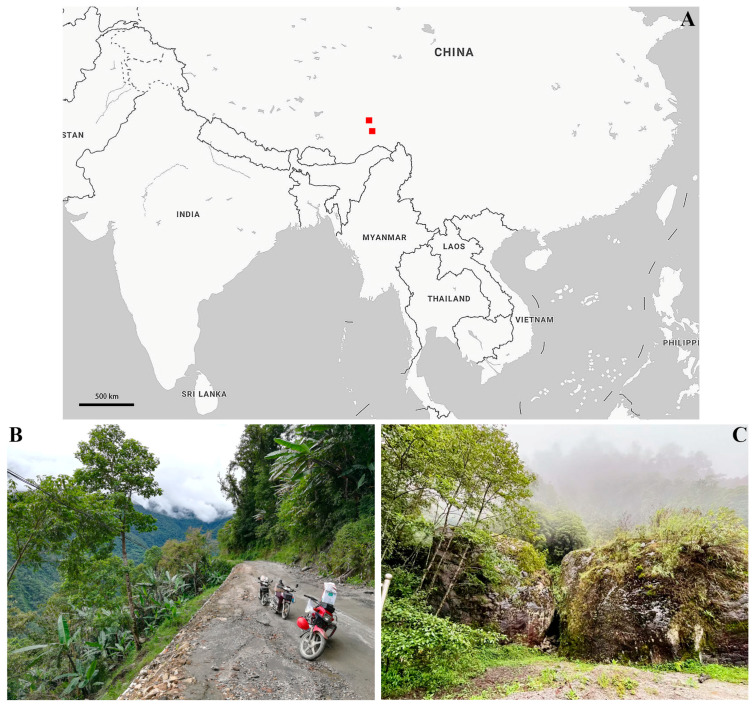
(**A**) The symbols “■” represents the collecting sites of *Luciargentis obesa* sp. nov. displayed on the Bing map; (**B**) a collecting site in Médog in 2017; and (**C**) a collecting site in Médog in 2023.

The examined specimens were collected using GYZ 450 W high-pressure mercury lamps (Yaming, Shanghai, China), and the collecting sites are showing in Figure 1. Morphological terminology in the descriptions was in accordance with Gozmány [3]. The wingspan was measured from the tips of the left and right forewings of fully well spread specimens. Slides of genitalia were prepared following Li [9]. Photographs of adults were captured using an M205A stereomicroscope, and genitalia photographs were taken using a DM750 microscope with Leica Application Suite software version 4.6 (Leica, Wetzlar, Germany). All images were processed with Photoshop CC (Adobe, San Jose, CA, USA). The type series of the new species are deposited at the Insect Collection of Nankai University (NKU), Tianjin, China, and at Liaocheng University (LCU), Liaocheng, China.

In this study, a total of 7 Lecithoceridae specimens were collected independently for molecular analysis, including 3 from *Luciargentis obesa* sp. nov., 1 from *Deltoplastis acutangulata*, 1 from *Thubana dialeukos*, 1 from *Halolaguna* sp., and 1 from *Thubana quadrilatera*. Genomic DNA was extracted from legs or partial body of dried specimens using Genomic DNA Extraction Kit (Tiangen Biotech, Beijing, China).

One mitochondrial marker (Cytochrome oxidase subunit 1 [COI]), and five nuclear markers (Carbamoyl phosphate synthetase domain protein [CAD], Elongation factor 1 alpha [EF-1α], Glyceraldhyde-3-phosphate dehydrogenase [GAPDH], ribosomal protein S5 [RpS5], and wingless) were amplified using polymerase chain reaction (PCR). The primers used sourced from previous studies [10,11,12,13]. When the published primers failed to amplify sequences, newly designed primers were used to obtain shorter fragments of the target regions in this study (Table 1). DNA amplification and sequencing protocols primarily followed those described by Wahlberg and Wheat [13]. The purified PCR products were directly sequenced using Sanger sequencing by Qingke Biotech (Beijing, China).

To construct a more comprehensive phylogenetic tree for Lecithoceridae, a 5350 bp dataset was downloaded from GenBank, which includes all available mixed COI and 6 nuclear gene sequences of 17 Lecithoceridae individuals [2,14,15]. This dataset included 1475 bp of COI, 850 bp of CAD, 691 bp of GAPDH, 925 bp of EF-1α, 600 bp of RpS5, 400 bp of wingless, and 407 bp of MDH (Table S1).

The sequences were manually edited in BioEdit v.7.2.5 [16] and examined using MEGA X software [17]. Each gene (COI, CAD, GAPDH, EF-1α, RpS5, wingless, and MDH) was aligned independently and concatenated into a dataset with a length of 5350 bp using the software PhyloSuite v1.2.2 [18]. We performed the phylogenetic reconstructions of Lecithoceridae species based on the concatenated dataset using maximum likelihood (ML) in IQ-TREE [19]. The Akaike Information Criterion (AIC) was used in PartitionFinder v2 [20] to select the best-fit model of sequence evolution for each locus alignmen (GTR + I + G for COI, GTR + G for CAD, GTR + I for MDH, SYM + I + G for wingless, RpS5, EF-1α, and GAPDH). The bootstraps were obtained using a rapid bootstrapping algorithm with 1000 replicates in the analysis of ML.

## 3. Results

### 3.1. Molecular Analysis Results

We obtained 3105 bp sequences for our specimen, including COI 648 bp for seven individuals, CAD 756 bp for two individuals, EF-1α 345 bp for seven individuals, GAPDH 522 bp for five individuals, RpS5 504 bp for five individuals, and wingless 330 bp for five individuals. These gene sequences generated in this study were deposited in GenBank under accession nos. PQ763539–PQ763545 (COI), PQ757619–PQ757625 (EF-1α), PQ757614–PQ757618 (GAPDH), PQ757626–PQ757630 (RpS5), PQ757612–PQ767513 (CAD), and PQ757631–PQ757635 (wingless) (Supplementary Materials).

**Figure 2:**
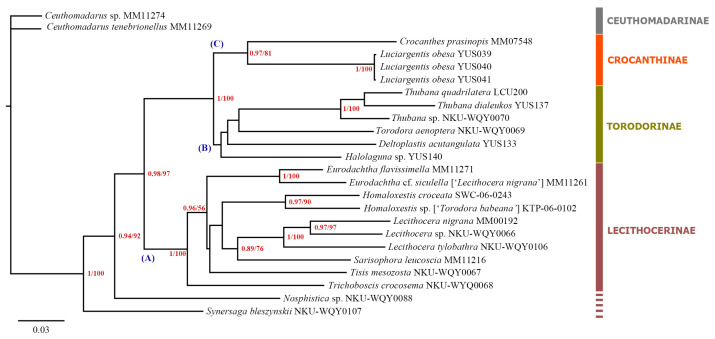
Phylogenetic tree of Lecithoceridae using 24 Lecithoceridae taxa based on a concatenated dataset of 5350 bp. The first support value for each node is Bayesian posterior probability (BPP), and the second number indicate bootstrap support value (BS). (A–C) indicate the three major clades in the phylogenetic tree.

The maximum likelihood (ML) tree and Bayesian inference (BI) tree were con-structed based on 24 exemplars representing 22 Lecithoceridae species, and the topological results shown in Figure 2. According to the phylogenetic tree topology, three major clades are recognized: A, B, and C. The clade A, representing the Lecithocerinae group, contains ten species from six genera; the clade B, representing the Torodorinae group, contains six species from four genera; and the clade C, representing the Crocanthinae group, contains two species including the newly described species, *Luciargentis obesa* sp. nov. The sister relationship between clade A and clade B + C was strongly supported (0.98/97%), and clade B was confirmed as the closest relative of clade C (1/100%). The branch of the new species is undoubtedly closer to the branch *Crocanthes prasinopis* (0.97/81%), and the two branches construct the clade C. Additionally, most intra-genus (e.g., *Thubana*, *Eurodachtha*, *Homaloxestis*, and *Lecithocera*) and intra-species (e.g., *Luciargentis obesa* sp. nov.) relationships were strongly supported (≥0.97/≥90%). The interspecific relationships of the clade B have not been well supported, but we resolved well the taxonomic affiliation of the new species which fall closest to the Crocanthinae rather than Torodorinae branch.

### 3.2. Morphological Results

#### 3.2.1. *Luciargentis* Yu and Wang, gen. nov.

Zoobank: urn:lsid:zoobank.org:act:6934232D-C8F4-405C-9944-EF9274588F7F

Type species: *Luciargentis obesa* Yu and Wang, sp. nov.

Gender: feminine.

Etymology: The genus name is derived from the Latin *luc*- and *argent*-, referring to the silvery marking of the forewing.

Diagnosis: *Luciargentis* gen. nov. belongs to the subfamily Crocanthinae. The new genus and another genus *Gonaepa*, possessing a basal plate of the gnathos in the male genitalia, differ from the remaining Crocanthinae genera which lacks the gnathos entirely (i.e., both the basal plate and the median process of the gnathos are absent). *Luciargentis* gen. nov. can be distinguished from the type species of the non-monophyletic genus *Gonaepa* Walker, *Gonaepa josianella* Walker, 1866 by the smooth antennae being longer than the forewing, in contrast to the strongly biciliate antennae that are nearly equal in length of the forewing in *G. josianella* (it should be noted that the type specimen of *G. josianella* is currently unavailable, and thus taxonomic comparisons are based solely on the original description) [21,22]. Moreover, the unique venation of *Luciargentis* gen. nov., characterized by stalked R_3_ + R_4_ + M_1_ and M_2_ + M_3_ + CuA_1_ + CuA_2_ in combination with its lanceolate forewing, serves as a diagnostic feature that distinguishes this new genus from all other genera within the subfamily Crocanthinae.

The possible synapomorphies of the genus includes the following: antenna smooth, distinctly longer than the forewing; forewing lanceolate, with slivery grey marking, R_5_ absent, M_1_ stalked with R_3+4_, M_2_, M_3_, CuA_1_, and CuA_2_ stalked; hindwing trapezoidal, M_2_ absent; abdominal tergites with zones of spiniform setae; male genitalia with a reduced gnathos possessing the basal plate but lacking the median process.

Key to the genera of the subfamily Crocanthinae:

1. Male genitalia with reduced gnathos (i.e., presence of a basal plate and absence of a median process) ……………………………………..…………………………………..... 2

- Male genitalia with gnathos entirely absent …………………………………………… 3

2. Antenna with long cilia; forewing subrectangular or triangular …...……… *Gonaepa*

- Antenna smooth; forewing lanceolate ………………………… *Luciargentis* gen. nov.

3. Labila palpus with sexual dimorphism ………………………………………………… 4

- Labial palpus with no sexual dimorphism ……………………………………………... 5

4. Hindwing unicolorous …………………………………………………..……… *Lamprista*

- Hindwing with similar maculation as forewing ………………………………… *Hanara*

5. Forewing with R_2_ usually free; hindwing usually unicolor ……………….... *Crocanthes*

- Forewing with R_2_ usually stalked with R_3+4_; hindwing with similar maculation as forewing ……………………………………………………………………………………. 6

6. Male labial palpus with second palpomere normal, third palpomere long or diversified ……………………………………………………………………………... *Aprosoesta*

- Male labial palpus with second palpomere extremely elongate, third palpomere very short or absent …………………………………………………………………… *Pacificulla*

The dichotomous key was partially adapted from Park [1].

#### 3.2.2. *Luciargentis obesa* Yu and Wang, sp. nov.

Zoobank: urn:lsid:zoobank.org:act:6B3D0238-E029-4ADE-B3CE-1DB0D5091131

Material examined: Holotype: ♂, China, Tibet, Médog, 2076 m, 29°40′ N, 95°30′ E, 28.vii.2018, leg. M.J. Qi, genitalia slide no. YS18209, in NKU. Paratypes: 1 ♂, China, Tibet, Médog, 880 m, 16.viii.2003, X.P. Wang and H.J. Xue leg., genitalia slide no. LSR12042, in NKU; 1 ♂, China, Tibet, Médog, 2089 m, 29°40′ N, 95°29′ E, 6.viii.2017, M.J. Qi leg., in NKU; 6 ♀♀, China, Tibet, Médog, 2089 m, 30°01′ N, 95°00′ E, 19.viii.2017, genitalia slide no. YS18210, in NKU; 1 ♀, China, Tibet, Bomi County, Tongmai Town, 2029 m, 30°06′ N, 95°05′ E, 15.viii.2018, M.J. Qi leg., genitalia slide no. YS18208, in NKU; 3 ♂♂, 3 ♀♀, China, Tibet, Médog, 1764 m, 29°20′ N, 95°22′ E, 17–18.vi.2023, S. Yu leg., genitalia slide nos. YUS039 ♂, YUS040 ♂, and YUS041 ♀, in LCU.

**Figure 3:**
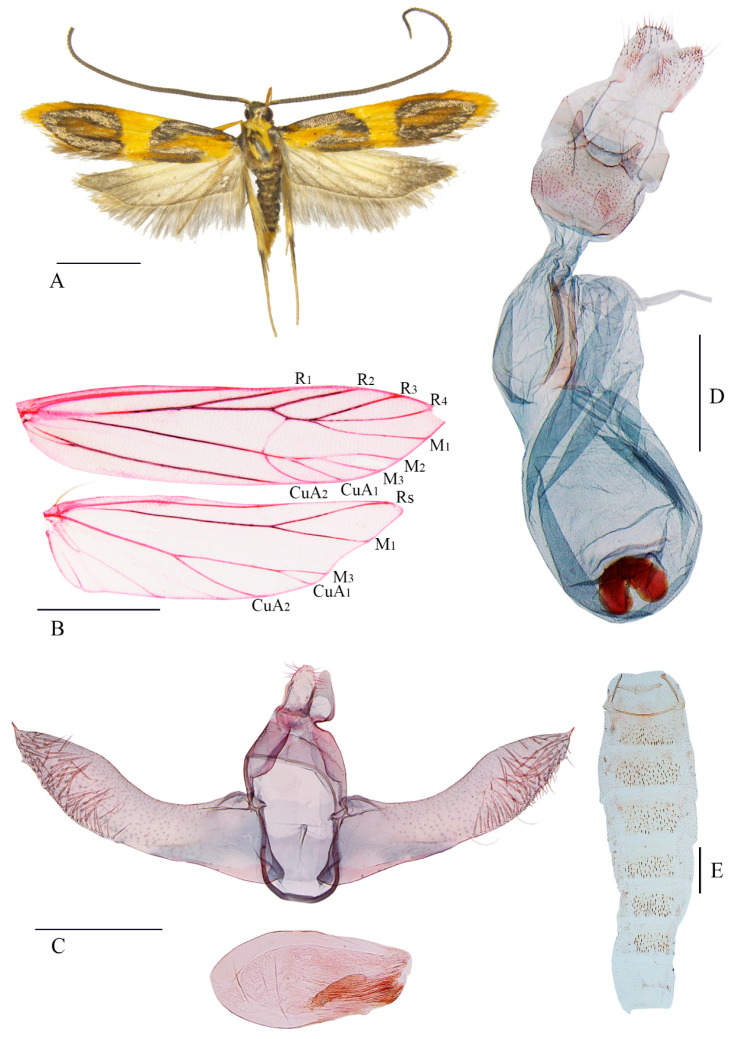
*Luciargentis obesa* Yu and Wang, sp. nov.: (**A**) adult, paratype, male; (**B**) wing venation, paratype, female, slide No. YS18208; (**C**) male genitalia, holotype, slide No. YS18209; (**D**) female genitalia, paratype, slide No. YS18210; (**E**) abdomen, paratype, slide No. YUS040. Scales: (**A**,**B**) = 2.0 mm; and (**C**–**E**) = 0.5 mm.

Description: Wingspan 15.5–17.5 mm (Figure 3A). Head greyish black, tinged with metallic luster. Antenna about 1.5 times length of forewing, greyish black except orange yellow dorsally on scape. Labial palpus orange yellow, third palpomere as long as the second. Thorax pale orange yellow; tegula slivery grey. Forewing lanceolate; ground color orange yellow, with two large slivery grey markings: first nearly U-shaped, upper branch along costal margin from base to basal 2/5, lower branch along mesial 1/3 of fold, yellowish brown on its concavity; second horseshoe-shaped, from about distal 2/5 along costal margin through termen to distal 1/4 of dorsum, dark yellowish brown on its concavity and greyish black at the open; a greyish black band along dorsum from basal 1/4 to middle; area between the first U-shaped marking and dorsum dark yellowish brown; fringe orange yellow except dark yellowish brown around tonus; cell closed; and R_1_, R_2_ free, R_3_, R_4_, M_1_ stalked and arising from anterior corner of discal cell, R_5_ absent, M_2_, M_3_, CuA_1_, and CuA_2_ stalked and arising from posterior corner of discal cell. Hindwing trapezoidal, orange white except grey on distal 1/3 and above dorsum; fringe grey, basal line orange white; cell open; and Rs and M_1_ stalked, M_2_ absent, M_3_ and CuA_1_ stalked, and CuA_2_ free (Figure 3B). Abdominal tergites with zones of spiniform setae (Figure 3E).

Male genitalia (Figure 3C). Uncus listric, parallel-sided in basal half, narrowed from middle to truncate apex, and setose distally on lateral sides. Gnathos with basal plate triangular. Valva wide at base, narrowed slightly to middle, widened at distal 1/3, thereafter narrowed to subacute apex, apex with a small spine; ventral margin nearly straight in basal 2/3, arched in distal 1/3; and sacculus wide at base, tapered to before middle of ventral margin. Vinculum narrow and U-shaped. Juxta shield-shaped, with an imbricate process at middle on posterior margin, obtuse on anterior margin. Aedeagus about 2/3 length of valva, stout, and ovate; vesica wrinkled and weakly sclerotized; and cornuti absent.

Female genitalia (Figure 3D). Papillae analis short. Apophyses posteriores slightly shorter than twice length of apophyses anteriores. Antrum weakly sclerotized, large, sub-rectangular, and spiculose, with a pair of digitiform lobes on posterior margin. Ductus bursae nearly as long as corpus bursae, posterior 1/3 narrowed, anterior 2/3 dilated, and as wide as corpus bursae, with a weakly sclerotized, longitudinal band; and ductus seminalis slender, arising from about middle of ductus bursae. Corpus bursae large elliptical; signum nearly semicircular, situated anteriorly.

Host: unknown.

Distribution (Figure 1): China (Tibet).

Etymology: The specific epithet is derived from the Latin *obesus*, referring to the stout aedeagus of the male genitalia.

## 4. Discussion

Crocanthinae currently comprises six genera, geographically restricted to the Australian, Oceanian, and Oriental regions [23]. Among the known genera, *Gonaepa* Walker, 1866 stands out as distinct, characterized by a reduced gnathos that features a basal plate but lacks a median process; in contrast, the other genera, *Aprosoesta* Turner, 1919, *Crocanthes* Meyrick, 1886, *Lamprista* Park, 2013, *Pacificulla* Park, 2013, and *Hannara* Park, 2013 completely lack a gnathos including both the basal plate and the median process. Additionally, *Gonaepa* is undoubtedly a compound group, exhibiting diverse wing shapes, patterns, and venation. For instance, *Gonaepa pyrochorda* Meyrick, 1910 and *G. phaeograpta* (Meyrick, 1931) have triangular forewings (see Park 2017: 88, figs 110, 112), while *G. dysthyma* Diaknoff, 1954 (see Park 2017: 88, fig 112-1), *G. ochrorhystima* Park, 2016 (see Park 2017: 113, fig 173), *G. nagaensis* Park, 2016, and *G. cordata* Park, 2016 (see Park 2017: 116, figs 178, 179) possess rectangular forewings [23]. The newly described genus, *Luciargentis* gen. nov. is most similar to *Gonaepa* in sharing the presence of a basal plate on the gnathos (see Park 2017: 100, figs 152, 153) [23]. Additionally, China previously recorded two subfamilies, Lecithocerinae, and Torodorinae. This study is the first to report presence of the subfamily Crocanthinae in China.

Wing venation is one of the most critical morphological characters used to distinguish genera in the current Lecithocerid classification system [3,24]. We found that Crocanthinae species share a wing venational synapomorphy (*Gonaepa* is not considered due to the unavailability of some morphological characteristics): forewing with R_5_ absent, M_3_ and CuA_1+2_ connate or stalked, hindwing with M_2_ absent. These wing venational characteristics support the classification of *Luciargentis* gen. nov. as part of the subfamily Crocanthinae.

On the other hand, the current concepts of Torodorinae and Crocanthinae, based solely on morphological characteristics, appear to overlap and failed to meet the demands of classification. According to Park et al. [7], the synapomorphy that distinguishes Crocanthinae from Torodorinae is the reduced gnathos, which is either completely absent or lacks a median process. However, some Torodorinae species also exhibit this feature, such as *Xenotodorodor stygioxanthus* Sterling, Lees and Grundy, 2023. Yu et al. [25] also noted that twelve species of *Torodora* (the type genus of Torodorinae) have a reduced gnathos with the median process absent. Additionally, as noted by Sterling et al. [26], further work is needed to confirm whether Torodorinae is a monophyletic group without including all or part of Crocanthinae. The phylogenetic tree in this study also demonstrates that the subfamilies Torodorinae and Crocanthinae are closest in the topology (1/100%). Furthermore, we find the placement of *Luciargentis* within the subfamily Crocanthinae convincing, as evidenced by the high clade node value of *Luciargentis obesa* + *Crocanthes prasinopis* (0.97/81%), which also satisfies the current research results and concepts of the subfamilies.

## Figures and Tables

**Table 1:** Designed primers used in this study.

Gene Region	Forward Primer (5′ to 3′)		Reverse Primer (5′ to 3′)	
EF-1α/elongation factor-1a	EF-1α-F	CCYGCCAAYATCACCACTGAAG	EF-1α-R	AGAGGHGGGAACTCYTGGAAGGA
GAPDH/Glyceraldehyde-3-phosphate dehydrogenase	GAPDH-F	TCACTTGGAVGGTGGHGCCAAGAA	GAPDH-R	AGAGAGATACCAGCDGCAGCATC
CAD/carbamoyl phosphate synthetase domain protein	CAD-F	AGTTTRGACTACTGTGTAGTTAAAATA	CAD-R	TGATAAAATAACGCCATCAGGA
MDH/cytosolic malate dehydrogenase	MDH-F	TGTTGTCATGGAGCTTGCAGATT	MDH-R	CCCATATAACAACATTCTTWACATCC
RpS5/ribosomal protein S5	RpS5-F	GCAGCATGGCCGTCGATAACAT	RpS5-R	TTGATGAACCCTTGGCAGCATTAAT
wingless	wingless-F	TGCACAGTGAAAACTTGCTGGAT	wingless-R	GTTACACCTTTCCACAACGAACATG

## Data Availability

All the sequences used in this study were accessed through the GenBank database and the accession numbers are listed in Table S1. Morphological specimens were deposited at the Insect Collection of Nankai University (NKU), Tianjin, China, and at Liaocheng University (LCU), Liaocheng, China.

## Supplementary Materials

The following supporting information can be downloaded at: https://www.mdpi.com/article/10.3390/insects16030242/s1, Table S1: List of specimen information used for this study.
